# Glucose Metabolism in Cancer: The Warburg Effect and Beyond

**DOI:** 10.1007/978-3-030-65768-0_1

**Published:** 2021

**Authors:** Sminu Bose, Cissy Zhang, Anne Le

**Affiliations:** Division of Hematology and Oncology, Department of Medicine, Columbia University Medical Center, New York, NY, USA; Department of Pathology, Johns Hopkins University School of Medicine, Baltimore, MD, USA; Department of Biology, Johns Hopkins University Krieger School of Arts and Sciences, Baltimore, MD, USA; Department of Pathology and Oncology, Johns Hopkins University School of Medicine, Baltimore, MD, USA; Department of Chemical and Biomolecular Engineering, Johns Hopkins University Whiting School of Engineering, Baltimore, MD, USA

**Keywords:** Glucose metabolism, Warburg effect, Glycogenolysis, Gluconeogenesis, Cancer metabolism

## 1 Introduction

Otto Warburg observed a peculiar phenomenon in 1924, unknowingly laying the foundation for the field of cancer metabolism. While his contemporaries hypothesized that tumor cells derived the energy required for uncontrolled replication from proteolysis and lipolysis, Warburg instead found them to rapidly consume glucose, converting it to lactate even in the presence of oxygen [1]. The significance of this finding, later termed the Warburg effect, went unnoticed by the broader scientific community at that time. The field of cancer metabolism lay dormant for almost a century awaiting advances in molecular biology and genetics, which would later open the doors to new cancer therapies [2, 3].

## 2 The Warburg Effect

### 2.1 Otto Warburg’s Early Studies of Normal Cellular Respiration

Warburg began his forays into research studying the oxygen consumption of sea urchin eggs, finding that the rate of respiration increased severalfold after fertilization. He went on to further describe two processes that were crucial to cellular glucose metabolism: respiration and fermentation [4].

Most differentiated cells metabolize glucose through the tricarboxylic acid (TCA) cycle under aerobic conditions. They then undergo oxidative phosphorylation to generate ATP (between 32 and 34 ATP molecules per glucose molecule) [5] (Fig. 1). While glycolysis produces two net molecules of ATP per one molecule of glucose, the majority of ATP production occurs during the TCA cycle and oxidative phosphorylation. During these latter steps of respiration, the pyruvate molecule produced in glycolysis undergoes a series of reactions in the presence of oxygen. Without the presence of oxygen, cells undergo fermentation or anaerobic glycolysis, shunting the resultant pyruvate molecules to lactate production.

### 2.2 The Warburg Effect Is a Prominent Feature of Cancer Cell Metabolism

In 1927, Warburg studied the processes of respiration and fermentation in tumor cells. According to normal cellular respiration, glucose is converted to pyruvate, which then enters the TCA cycle to undergo oxidative phosphorylation in the presence of oxygen, and there should be minimal lactate production. However, in his in vivo and ex vivo studies, Warburg observed an increased glucose uptake and increased lactic acid production in tumor cells as compared to normal cells, even in the presence of oxygen [6]. This phenomenon, the metabolism of glucose to lactate despite the presence of adequate oxygen, is called the Warburg effect or aerobic glycolysis (Fig. 1).

For Warburg, several questions remained unanswered, including why cancer cells would inefficiently shunt glucose-derived pyruvate to lactate production instead of to the TCA cycle, which would result in significantly higher ATP production. Warburg hypothesized that the lactate production in cancer cells was due to the impairment of oxidative phosphorylation caused by mitochondrial damage [7].

There was a debate surrounding this theory with disagreement arising particularly from Sidney Weinhouse, one of Warburg’s contemporaries. Using isotope tracing [8], Weinhouse’s experiments showed that the rates of oxidative phosphorylation in both normal cells and tumor cells are similar, suggesting that the mitochondria of tumor cells are intact [9]. Rather, tumor cells in oxygen-rich environments utilize both aerobic glycolysis and oxidative phosphorylation to sustain their rapid rates of proliferation. Only in hypoxic environments, such as the tumor core, do the rates of lactic acid production by anaerobic glycolysis overtake oxidative phosphorylation as the primary source of energy [10].

### 2.3 The Biochemical Nature and Clinical Significance of the Warburg Effect

Examination of the underlying biochemical processes elucidated possible reasons for why cancer cells paradoxically undergo aerobic glycolysis, a process yielding less ATP than oxidative phosphorylation per cycle. For example, given the inefficiency of ATP production in the Warburg effect, there are likely differences in the kinetics of aerobic glycolysis and oxidative phosphorylation, which have led to cancer cells promoting aerobic glycolysis. Demetrios et al. found that, in the Warburg effect, the flux of glucose to lactate is up to 100 times faster than through the TCA cycle resulting in similar amounts of ATP production over the same time [11]. Even when oxidative phosphorylation is actively continuing, aerobic glycolysis will see much higher glucose flux [12].

To further understand a cancer cell’s dependence on aerobic glycolysis, it is necessary to revisit one of the hallmarks of cancer—rapid proliferation supported by strong anabolism. Tumor cells need not only ATP but also anabolic metabolism to accumulate a large amount of biomass to sustain their growth. The Warburg effect via multiple glycolytic intermediates provides a carbon source which contributes to the nucleotide, fatty acid, and amino acid synthesis pathways [13]. For example, glucose-6-phosphate (G6P) becomes partially oxidized via the pentose phosphate pathway (PPP) to generate NADPH and nucleotide components. In addition to the PPP, NADPH is also generated with the shunting of 3-phosphoglycerate out of the glycolytic pathway and into the serine and glycine biosynthesis pathway [14]. NADPH is a reducing equivalent, which is then further used for lipid biosynthesis [15, 16]. In addition, phospholipid biosynthesis is enabled by the conversion of dihydroxyacetone phosphate (DHAP) to glycerol-3-phosphate [17], and fructose-6-phosphate (F6P) enters the hexosamine pathway to support protein posttranslational modification [18].

Two other clinically significant hallmarks of cancer, the evasion of apoptotic cell death and the ability to metastasize, may provide additional reasons behind the upregulation of aerobic glycolysis in cancer. Anoikis is a type of apoptosis that is a consequence of reactive oxygen species (ROS) accumulation in the setting of the detachment of a cell from the extracellular matrix [19]. When this detachment happens for a cancer cell, however, anoikis is inhibited because the Warburg effect reduces mitochondrial ROS production by decreasing the flow of pyruvate into oxidative phosphorylation [20]. Resistance to apoptosis in the setting of matrix detachment is essential to the metastatic spread of tumor cells.

The Warburg effect has clinical utility as well. One ubiquitous application is the use of positron-emission tomography (PET) imaging in oncology, which has become indispensable in the detection of tumors and the monitoring of the response of existing cancer to therapeutic intervention. PET is an exploitation of the high rate of glycolysis in cancer cells as it uses a radiolabeled glucose analog, [^18^F]fluoro-2-deoxy-d-glucose (FDG), which accumulates in tumor cells due to their rapid uptake of glucose. Another developing application of the Warburg effect is the use of gene expression profiles linked to glycolysis to determine prognosis. Tools in both lung adenocarcinoma and triple-negative breast cancer have shown that glycolytic phenotypes are generally associated with worse patient survival [21–23].

### 2.4 Metabolic and Genetic Reprogramming Underlying the Warburg Effect

With current advances in genetics and molecular biology, much of the past several decades of cancer research have been consumed by characterizing the genetic alterations, which lead to the development of cancers. However, cancer cells need not only a genetic switch but also metabolic building blocks and energy sources to undergo rapid proliferation. The recognition of the importance of energy sources allowed for the resurgence of cancer metabolism as a field that is closely related to tumor genetics. It is now understood that the metabolic reprogramming underlying the Warburg effect is driven by several oncogenes and tumor suppressors.

Some of the identified oncogenes, namely protein kinase B (PKB/AKT), phosphoinositide 3-kinase (PI3K), Ras, and Von Hippel-Lindau (VHL), act via the protein hypoxia-inducible factor 1α (HIF-1α), resulting in the non-hypoxic expression of HIF-1α. In normal cells, HIF-1α becomes stabilized in a hypoxic environment to form a transcription factor involved in promoting glycolysis and suppressing oxidative phosphorylation [24]. HIF-1α, when present, upregulates glucose transporter 1 (GLUT1) to promote the retention of glucose inside cells in addition to upregulating hexokinase 2 (HK2), the enzyme which catalyzes the first committed step of glycolysis [25]. Typically, when oxygen is present, HIF-1α degrades in a concentration-dependent manner. In tumor cells, however, even in the presence of oxygen, high AKT and mechanistic target of rapamycin (mTOR) oncogenic activity promote HIF-1α expression, leading to persistent transcription of the enzymes driving glycolysis and lactate production.

Other oncogenic pathways have been found to work independently of HIF-1α to promote aerobic glycolysis as well, namely the activation of oncogenes such as *MYC*, *Ras*, and *AKT* and the deactivation of tumor suppressors such as TP53. Like HIF-1α, *MYC* directly upregulates GLUT and HK2. The loss of TP53 function also upregulates GLUT expression. Additionally, TP53 deactivation indirectly leads to increased glycolysis. Without TP53 expression, TP53-induced glycolysis and apoptosis regulator (TIGAR), a protein, which causes shunting of glucose to the PPP, is no longer upregulated, resulting in a greater flux of glucose through the glycolytic pathway [26].

## 3 Heterogeneity in Glucose Metabolism

Aerobic glycolysis is not consistent across tumor types or even within a single tumor’s microenvironment (Fig. 2). Examination of different tumor types revealed that the balance between aerobic glycolysis and oxidative phosphorylation could be very different. In a study evaluating the variability of metabolic gene expression across multiple different tumor types, it was found that there was an upregulation of genes related to oxidative phosphorylation in ovarian, lymphoma [27], leukemia, and lung cancers, whereas the opposite was true in thyroid, colon, pancreatic [28], and renal cancers [29, 30]. It is thought that the variable activation of different oncogenes such as *RAS*, *AKT*, and c-*MYC* is the driver behind these differences [31–35].

These differences in metabolism can be seen even in cells within the same tumor [36, 37]. Sometimes, these differences result from variations in the tumor microenvironment leading to metabolic flexibility, the ability of cancer cells to change their bioenergetic pathways according to available nutrients [38, 39]. One important resource is oxygen, which can vary significantly with the aberrant vascularization of tumors. As demonstrated in HeLa cells in hypoxia, there was an observed decrease in ATP derived from oxidative phosphorylation to just 29% compared to 79% in normoxia [40]. However, in a study by Le et al., it was shown that a subpopulation of cancer cells under hypoxia still exhibited expressions of genes related to mitochondrial function and maintained their oxidative phosphorylation and tumorigenicity [37]. These results suggest that respiration, even when there is an oxygen shortage, may be necessary for tumorigenicity, which does not depend on the Warburg effect alone and is not reduced as a result of the maintenance of respiration under hypoxic conditions. There may also be differences rooted in the type of tumor cells within the microenvironment—cancer stem-cell-like cells (CSCs) versus more differentiated tumor cells. A recent study found that 80% showed high levels of glucose uptake, and 20% showed low levels of glucose uptake [41]. This may have been due to the presence of both CSCs and differentiated cells within the studied population. Similarly, studies of small-cell lung cancer (SCLC) cells showed that the CSC subpopulation was metabolically less active and preferred oxidative phosphorylation rather than glycolysis to fulfill energy requirements [42].

## 4 The Role of Glycogen Metabolism and Gluconeogenesis in Tumor Growth

### 4.1 Glycogen Metabolism Is Upregulated in Several Cancers

Glycolysis is not the only component of glucose metabolism, which plays a significant role in tumor growth. Glycogenolysis, the process by which glycogen is converted to glucose-1-phosphate (G1P) and then to G6P to enter the glycolytic pathway, provides another energy source for tumors in the face of nutrient stress (Fig. 2). Glycogen metabolism, although studied far less than glycolysis by cancer researchers, is upregulated in many cancer types, including renal, breast, bladder, uterine, ovarian, skin, and brain cancers. However, the glycogen content of cancer cells was found to be not associated with the rate of replication [43]. Renal cell carcinoma, which classically has clear cells on histology, appears this way due to high glycogen content.

Advances in tumor genetics have allowed for the characterization of tumor-suppressor genes and oncogenes, which have driven these changes in glycogen metabolism in tumor cells. The over-expression of the oncogene Rab25 has been demonstrated as a driver in increasing cellular glycogen stores via the AKT pathway [44]. In bladder cancer, the glycogen debranching enzyme AGL has been identified as a tumor suppressor. Additionally, deactivation of AGL leads to the accumulation of abnormal glycogen stores and promotes tumorigenesis in xenograft models [45]. Given this, Guo-Min Shen and colleagues studied glycogen metabolism in the setting of hypoxia. It was noted that glycogen accumulated in breast cancer cells after 24 and 48 h under hypoxia due to HIF-1α induction of protein phosphatase 1 regulatory subunit 3C (PPP1R3C), a glycogen synthase [46]. Later studies demonstrated that glycogen synthesis promotes cancer cell survival in the setting of hypoxic conditions [47]. Both glycogenolysis and glycogen synthesis enzymes appear to be upregulated by tumor cells with HIF-1α dependence, including UTP:glucose-1-P uridylyltransferase 2 (UGP2), phosphoglucomutase (PGM), 1,4-alpha-glucan branching enzyme (GBE), glycogen synthase 1 (GYS1), and PPP1R3C [48]. In vivo studies of suppression of glycogen synthase kinase 2 (GSK2) activity demonstrated a reduction in prostate tumor growth [49]. Glycogen metabolism is an important target of therapy given that cancer cells can utilize glycogen as an energy source even during nutrient deficiency due to poor angiogenesis [50, 51].

### 4.2 Upregulation of Gluconeogenic Enzymes in Cancer

Gluconeogenesis is the process of generating glucose from carbon substrates that are not carbohydrates. There are two gluconeogenic enzymes that play important roles in cancer metabolism: phosphoenolpyruvate carboxykinase 1 (PCK1) and phosphoenolpyruvate carboxykinase 2 (PCK2). It has been demonstrated that TP53 inhibits both enzymes, meaning that the loss of TP53 upregulates these enzymes and gluconeogenesis [52]. It was also observed that the inhibition of mTOR in hepatocellular carcinoma and renal cell carcinoma cells directs the glycolytic flux towards lactate and gluconeogenesis with resultant tumor cell death via the downregulation of PCK1 [53].

## 5 Success and Failures of Targeting Glucose Metabolism for Cancer Therapy

### 5.1 Therapies Targeting Glycolysis and the Warburg Effect

As discussed previously, over the latter half of the twentieth century, advances in molecular biology and the identification of oncogenes and tumor suppressors drew the attention of much of the anticancer therapeutic efforts. It is true that genetic alterations drive uncontrolled replication in cancer cells, but it is also important to recognize that a cancer cell is still dependent on nutrient availability. In the past two decades, there has been an upsurge in efforts to exploit the addiction of cancer cells to glucose and the Warburg effect for cancer treatment [54]. Several enzymes in the glycolytic pathway have been targeted, some showing tumoricidal effects in vitro and in vivo (Fig. 3). Unfortunately, there has been little clinical success given that glycolysis is crucial to the glucose metabolism of normal cells as well. Thus, the focus should be on targeting those elements of aerobic glycolysis, which are more upregulated in cancer.

Glucose transporters (GLUT1–4) are upregulated in tumor cells by MYC and HIF-1α. Previous attempts with small-molecule inhibitors of GLUT1 have seen in vitro tumoricidal effects in a renal cell carcinoma cell line [55] and hepatocellular carcinoma cell line [56]. However, GLUT1 is a prevalent glucose transporter in normal cells as well, which would likely preclude clinical success. Homozygous *Glut1* deletion is embryonically lethal in mice, and heterozygous deletion causes impaired motor activity and seizures [57]. A GLUT1 inhibitor called silibinin failed to demonstrate any reduction in prostate-specific antigen, a well-known biomarker for prostate cancer, in a phase I clinical trial and was associated with significant side effects [58].

Hexokinase phosphorylates glucose to glucose-6-phosphate in the first committed step of glycolysis. Hexokinase 2 (HK2) is mostly expressed in cancer cells and is the primary hexokinase to function in tumors, so it is another potential therapeutic target. Experiments in which HK2 was systemically deleted have shown to be well tolerated in mice [59]. A glucose analog that competitively inhibits G6P isomerase in order to inhibit the phosphorylation of glucose, 2-deoxyglucose, has been studied in a phase I clinical trial in combination with radiation therapy with good toleration in glioblastoma multiforme [60, 61]. However, a HK inhibitor called lonidamine failed to show any benefit in two phase III randomized clinical trials [58].

Phosphofructokinase (PFK) is the enzyme which catalyzes the second committed step in glycolysis, the conversion of fructose-6-phosphate to fructose-1,6-bisphosphate (F1,6-BP). Although inhibiting PFK directly is not possible since it is crucial to glycolysis in normal cells, it may be feasible to target it indirectly. PFK is strongly allosterically activated by fructose-2,6-bisphosphate (F2,6-BP). F2,6-BP is activated by another protein, 6-phosphofructo-2-kinase/fructose-2,6-biphosphatase 3 (PFKFB3), a target of HIF-1α. Attenuation of glycolysis was achieved in in vitro and in vivo studies with a small-molecule PFKFB3 inhibitor called 3-(3-pyridinyl)-1-(4-pyridinyl)-2-propen-1-one (3PO) [62]. PFKFB3 inhibitors were also shown to reduce tumor angiogenesis [63].

Glyceraldehyde 3-phosphate dehydrogenase (GAPDH) converts glyceraldehyde 3-phosphate to glycerate 1,3-bisphosphate with the production of NADH and is a promising target for anti-glycolytic therapy given the role of NADH in biosynthesis. The small-molecule pyruvate analog, 3-bromopyruvate, is a nonselective inhibitor of GAPDH and has been shown to inhibit tumor oxidative phosphorylation and glycolysis with good preclinical efficacy [64, 65]. However, there is concern for toxicities such as burning sensation with intravenous infusion and there are no ongoing clinical trials with this compound [66].

In seeking a target that was more unique to cancer cell metabolism and central to the Warburg effect, Le et al. focused on lactate dehydrogenase A (LDHA) which reciprocally mediates the redox-coupled conversion between lactate with NAD+ and pyruvate with NADH [67, 68]. Elevated expression level of LDHA is a hallmark of many types of tumors, including squamous head and neck cancer, colorectal cancer, and non-small cell lung cancer [69–71]. By perturbing the NADH/NAD+ ratio, a small-molecular inhibitor of LDHA called FX-11 was shown to increase reactive oxygen species in tumor cells with subsequent cell death in not only in vitro studies but also pancreatic and lymphoma xenografts [72–74].

Several other LDHA inhibitors, such as gossypol, galloflavin, and *N*-hydroxyindole-based inhibitors, were tested in preclinical settings [72, 75–78]. Among them, gossypol (AT-101), a nonselective inhibitor of LDH, was tested in phase I and phase II clinical trials targeting glioblastoma (NCT00390403, NCT00540722), small-cell lung cancer, and prostate cancer [79, 80]. Despite active investigations for developing LDH inhibitors, there is still a clinical need for highly selective and efficient LDH inhibitors, as gossypol shows off-target effects such as the inhibition of NADH-dependent enzymes (e.g., GAPDH) [77]. Although compounds targeting lactate metabolism have not yet been approved, it is clear that LDH-targeting strategies are promising approaches for cancer therapy.

On a macro level, dietary changes to limit glucose availability to tumor cells have also been studied. For example, ketogenic therapy, a diet with severe carbohydrate restriction, has been shown to sensitize gliomas and glioblastoma to chemoradiation therapy, reduce oxidative stress, and downregulate angiogenic proteins [81]. The success of this therapy may lie in the relative metabolic inflexibility of neuronal cells and their addiction to glucose.

### 5.2 Therapies Targeting Glycogenolysis and Glycogen Synthesis Have Shown Promising Results

Significantly fewer therapies targeting glycogen metabolism have been developed (Fig. 3). Lee et al. inhibited glycogen phosphorylase in a pancreatic cell line with a compound called CP-320626, leading to tumor cell death with no effect on normal human fibroblasts [82]. Flavopiridol, another glycogen phosphorylase inhibitor, had safe and modest efficacy in clinical trials with prostate cancer, renal cell carcinoma, and colorectal carcinoma [83–85]. However, flavopiridol is also a cyclin-dependent kinase inhibitor [86], so it is uncertain whether the antitumor effects were purely from glycogen phosphorylase inhibition. More recently, inhibition of glycogen synthase kinase (GSK)3β by AR-A014418 and SB-216763 in an esophageal squamous cell carcinoma cell line has resulted in attenuated tumor growth and induced apoptosis; thus GSK3β has emerged as a potential target [87]. Similar results were shown in sarcoma cell lines [88]. Given these promising results, further investigation of glycogenolysis and glycogen synthesis-targeting agents is warranted.

## 6 Conclusion

Currently, there are several challenges to metabolic cancer therapies. First, an understanding of the heterogeneity of metabolic phenotypes is only beginning to be established. Metabolic phenotypes likely vary based on tissue of origin, tumor microenvironment, primary versus metastatic tumors, and mutational differences. Second, there are limitations in translating in vivo mouse studies to clinical trials, as is evidenced by the lack of success in advancing metabolic inhibitors through clinical trials up until this point. Third, there is the potential for metabolic inhibitors to be overcome by the adaptation of tumors to new energy sources as well as their inherent metabolic flexibility. With renewed interest in cancer metabolism, the development of metabolic inhibitors will continue to grow, and it may be most effective to combine these therapies with other modalities of therapy in order to increase efficacy.

## Figures and Tables

**Fig. 1:**
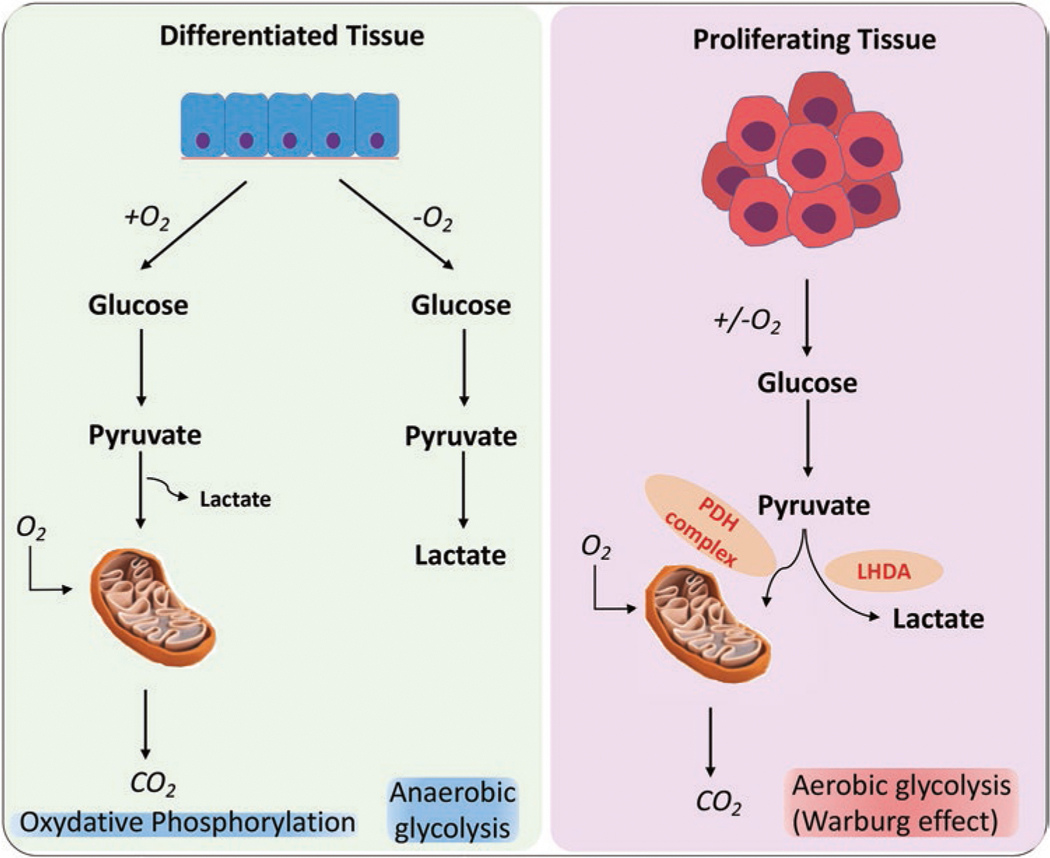
Respiration in normal differentiated tissue (left) in contrast with the Warburg effect in proliferating tissue (right)

**Fig. 2:**
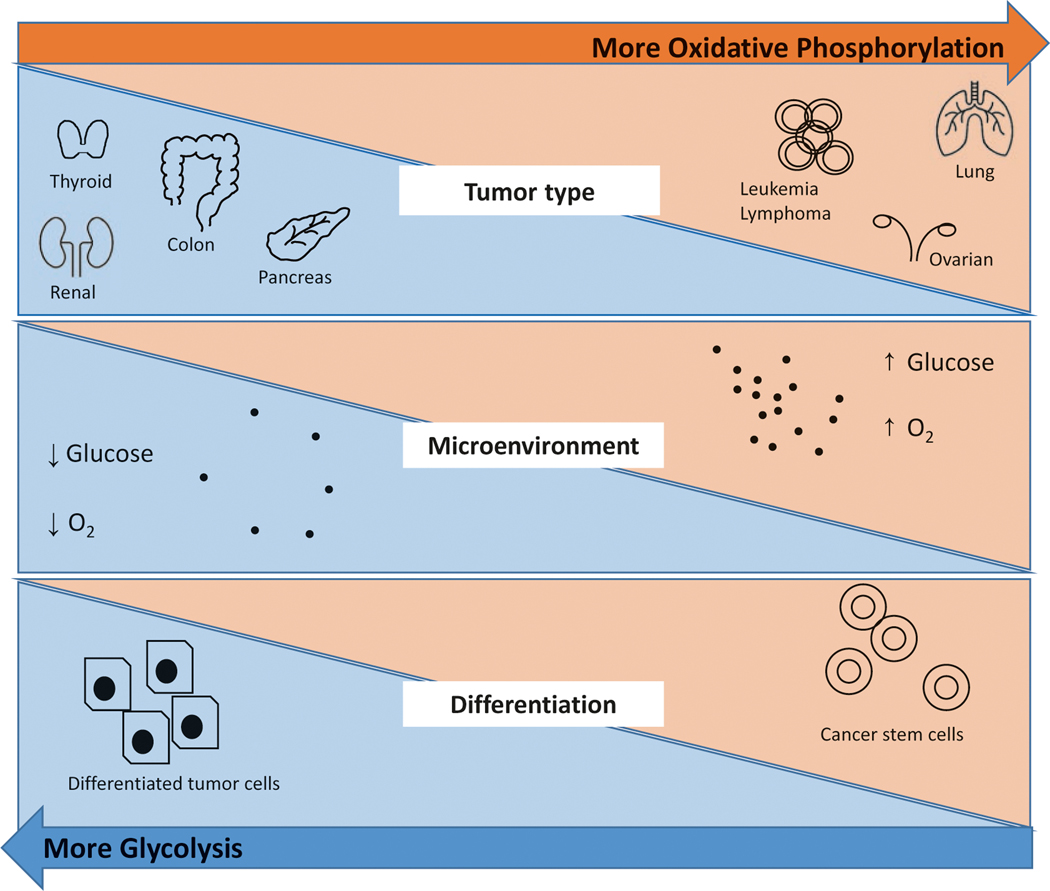
Heterogeneity in cancer glucose metabolism with respect to tumor type, tumor microenvironment, and differentiation

**Fig. 3:**
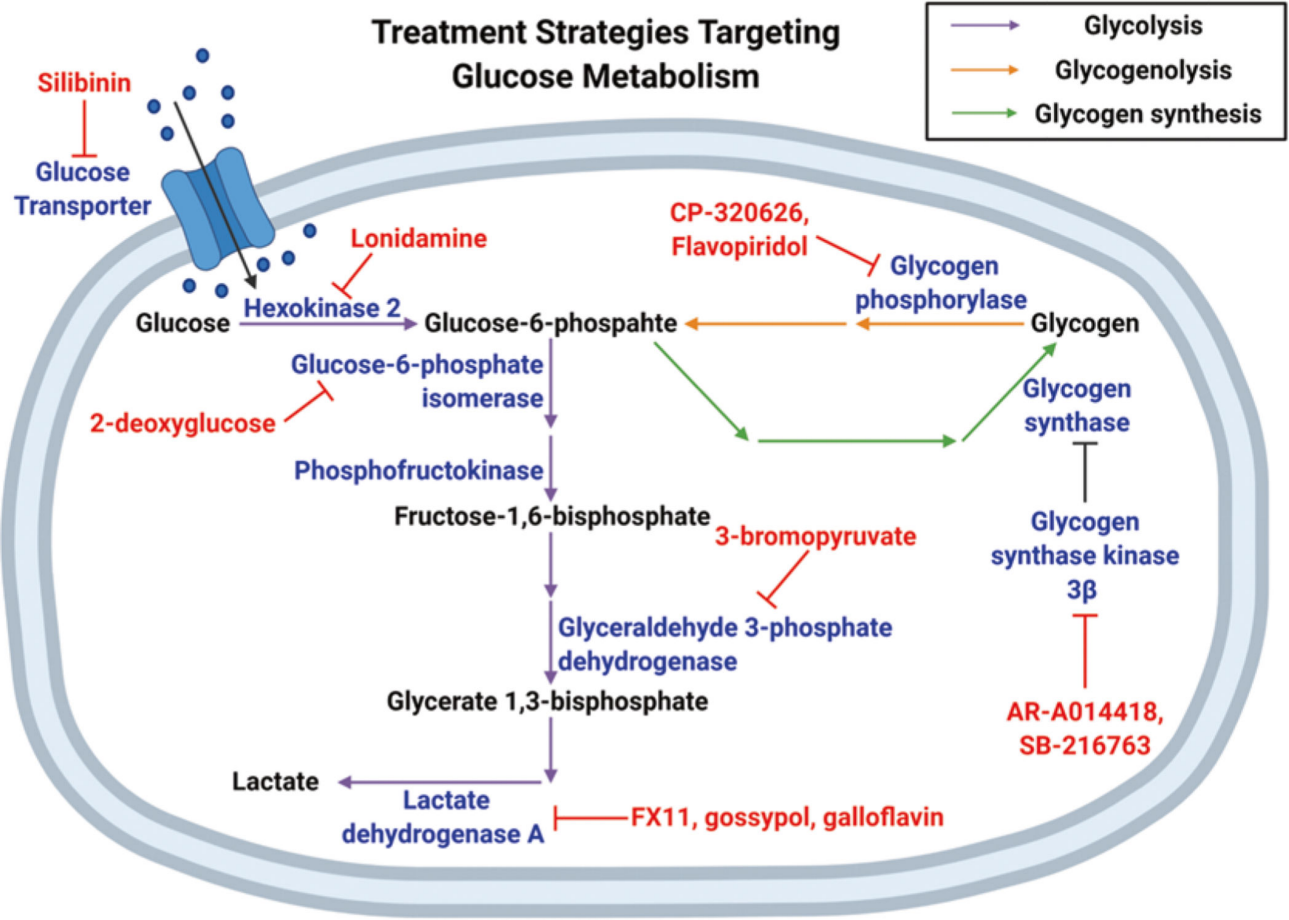
Current targets of cancer therapies directed at glucose metabolism

## Key Points

- Tumor cells exhibit an upregulation in glycolysis, glycogen metabolism, and gluconeogenesis as opposed to normal cells.
- Several oncogenes and tumor suppressors drive the metabolic reprogramming underlying the Warburg effect and other changes in glucose metabolism.
- There is heterogeneity in glucose metabolism across tumor types as well as within the tumor microenvironment.
- Numerous therapies targeting glucose metabolism have been developed but have yet to show success in clinical trials.

## Abbreviations

3PO: 3-(3-Pyridinyl)-1-(4-pyridinyl)-2-propen-1-one
AGL: Amylo-alpha-1, 6-glucosidase, 4-alpha-glucanotransferase
AKT: Also known as PKB, protein kinase B
ATP: Adenosine triphosphate
CP-320626: 5-Chloro-*N*-[(2S)-3-(4-fluorophenyl)-1-(4-hydroxypiperidin-1-yl)-1-oxopropan-2-yl]-1H-indole-2-carboxamide
CSC: Cancer stem-cell-like cell
DHAP: Dihydroxyacetone phosphate
F1,6-BP: Fructose-1,6-bisphosphatase
F2,6-BP: Fructose-2,6-bisphosphate
F6P: Fructose-6-phosphate
FDG: Fluoro-2-deoxy-d-glucose
FX-11: 3-Dihydroxy-6-methyl-7-phenylmethyl-4-propylnaphthalene-1-carboxylic acid
G1P: Glucose-1-phosphate
G6P: Glucose-6-phosphate
GAPDH: Glyceraldehyde 3-phosphate de-hydrogenase
GBE: 1,4-Alpha-glucan branching enzyme
GLUT: Glucose transporter
GSK2: Glycogen synthase kinase 2
GSK3β: Glycogen synthase kinase 3β
GYS1: Glycogen synthase 1
HIF-1α: Hypoxia-inducible factor 1α
HK2: Hexokinase 2
LDHA: Lactate dehydrogenase A
mTOR: Mechanistic target of rapamycin
NAD: Nicotinamide adenine dinucleotide
NADPH: Nicotinamide adenine dinucleotide phosphate
PCK1: Phosphoenolpyruvate carboxyki-nase 1
PCK2: Phosphoenolpyruvate carboxyki-nase 2
PET: Positron-emission tomography
PFK: Phosphofructokinase
PFKFB3: 6-Phosphofructo-2-kinase/fructose-2,6-biphosphatase 3
PGM: Phosphoglucomutase
PI3K: Phosphoinositide 3-kinase
PPP: Pentose phosphate pathway
PPP1R3C: Protein phosphatase 1 regulatory subunit 3C
ROS: Reactive oxygen species
SCLC: Small-cell lung cancer
TCA: Tricarboxylic acid
TIGAR: TP53-induced glycolysis and apoptosis regulator
TP53: Tumor protein 53
UGP2: UTP:glucose-1-P uridylyltransferase 2
VHL: Von Hippel-Lindau
